# Oncostatin M Counteracts the Fibrotic Effects of TGF-β1 and IL-4 on Nasal-Polyp-Derived Fibroblasts: A Control of Fibrosis in Chronic Rhinosinusitis with Nasal Polyps?

**DOI:** 10.3390/ijms23116308

**Published:** 2022-06-04

**Authors:** Florent Carsuzaa, Émilie Béquignon, Matthieu Bainaud, Jean-François Jégou, Xavier Dufour, Jean-Claude Lecron, Laure Favot

**Affiliations:** 1 Laboratoire Inflammation Tissus Epithéliaux et Cytokines (LITEC), UR15560, Université de Poitiers, 86000 Poitiers, France; matthieu.bainaud@univ-poitiers.fr (M.B.); jean-francois.jegou@univ-poitiers.fr (J.-F.J.); xavier.dufour@chu-poitiers.fr (X.D.); jean-claude.lecron@univ-poitiers.fr (J.-C.L.); laure.favot@univ-poitiers.fr (L.F.); 2 Service ORL, Chirurgie Cervico-Maxillo-Faciale et Audiophonologie, Centre Hospitalier Universitaire de Poitiers, 86000 Poitiers, France; 3 Service ORL et Chirurgie Cervico-Faciale, Centre Hospitalier Intercommunal de Créteil, 94000 Créteil, France; emilie.bequignon@chicreteil.fr; 4 Laboratoire INSERM UMR955 Eq13, Institut Mondor de Recherche Biomédicale, 94000 Créteil, France; 5 Service Immunologie et Inflammation, Centre Hospitalier Universitaire de Poitiers, 86073 Poitiers, France

**Keywords:** chronic rhinosinusitis, cytokines, inflammation, nasal polyps, fibroblasts, fibrosis

## Abstract

Chronic rhinosinusitis with nasal polyps (CRSwNP) is associated with inflammation and tissue remodeling including myofibroblasts differentiation and extracellular matrix (ECM) deposition mediated by TGF-β1 and IL-4. Oncostatin M (OSM) is a cytokine involved in fibrotic processes in other cellular subtypes. We investigated the mechanisms of action of OSM in the fibrosis process associated with CRSwNP. The expression of IL-4, OSM and TGF-β1 was assessed by RT-qPCR. Primary human cultures of nasal-polyp-derived fibroblasts were established and stimulated by TGF-β1 and/or IL-4 and/or OSM. The expression of ECM components and αSMA was determined by RT-qPCR and Western blot. TGF-β1-Smad3 signaling was investigated by immunofluorescence. TGF-β1, IL-4 and OSM as well as αSMA were overexpressed in nasal polyps when compared to noninflammatory nasal mucosa. In TGF-β1-stimulated nasal-polyp-derived fibroblasts, ECM genes and αSMA gene and protein were overexpressed, as well as αSMA in IL-4-stimulated fibroblasts. OSM counteracted the profibrotic effect of TGF-β1 on ECM components and αSMA. TGF-β1-induced nuclear translocation of Smad3 was completely reversed by OSM. OSM counteracts the profibrotic effect of IL-4 and also TGF-β1, by inhibiting the nuclear translocation of Smad3. We suggest OSM could be an efficient tool to protect against fibrosis in CRSwNP.

## 1. Background

Chronic rhinosinusitis with nasal polyps (CRSwNP) is an inflammatory disease that affects 4% of the general population. Symptoms are related to the presence of polyps in the nasal cavities and paranasal sinuses and can include hyposmia or anosmia, nasal obstruction, discharge and facial pain [1]. Nasal polyps are characterized by soft-tissue edema, overgrowth of the nasal mucosa and increased fibrosis. Although its pathophysiology has not yet been clearly identified, chronic type 2 inflammation remains a key factor in the formation of nasal polyps [2]. A remodeling profibrotic process takes place in the nasal polyps including a myofibroblast differentiation of fibroblasts, accumulation of extracellular matrix (ECM) components, of which the most described are collagen 1A1 and 1A2, and also infiltration of inflammatory cells [3].

Regarding fibrosis, the transforming growth factor-beta-1 (TGF-β1), produced by fibroblasts and immune cells, induces a myofibroblast differentiation and accumulation of ECM proteins [4]. Interleukin-4 (IL-4), a cytokine mainly involved in the maintenance of type 2 immune profile, the production of immunoglobulins and eosinophil migration [5], is also involved in fibrosis with a profibrotic role [6]. TGF-β1 and IL-4 are the two most widely described cytokines involved in the profibrotic process in CRSwNP [7].

Oncostatin M (OSM) is a multifunctional cytokine belonging to the IL-6 family, produced by dendritic cells, monocytes/macrophages, neutrophils and T cells. OSM is overexpressed in nasal polyps compared to nonpolypoid nasal mucosa in chronic rhinosinusitis [8]. On human epithelial cell cultures from nasal polyps, OSM causes an alteration of the epithelial barrier by decreasing tight junctions [8]. OSM also plays an important role in fibrotic diseases such as systemic scleroderma, rheumatoid arthritis, interstitial pulmonary fibrosis as well as excessive scarring [9,10,11,12] and can have a pro or an antifibrotic effect depending on the model. At a time of an increased use of biotherapies for CRSwNP, OSM could be a new target for new therapeutic treatments.

In the present study, we investigated the putative role of OSM in the fibrosis process associated with the pathophysiology of CRSwNP. After analyzing the cytokine receptor expression patterns in nasal-polyp-derived fibroblasts, we measured OSM mRNA expression in nasal polyps. The ability of OSM, associated or not with TGF-β1 and IL-4, to modulate the expression of ECM mRNA and proteins was studied in vitro on CRSwNP-derived fibroblasts. Finally, we investigated the modulation of the TGF-β1-Smad3 signaling pathway by OSM.

## 2. Results

### 2.1. TGF-β1, IL-4 and OSM as Well as αSMA Were Overexpressed in Nasal Polyps

**Figure 1:**
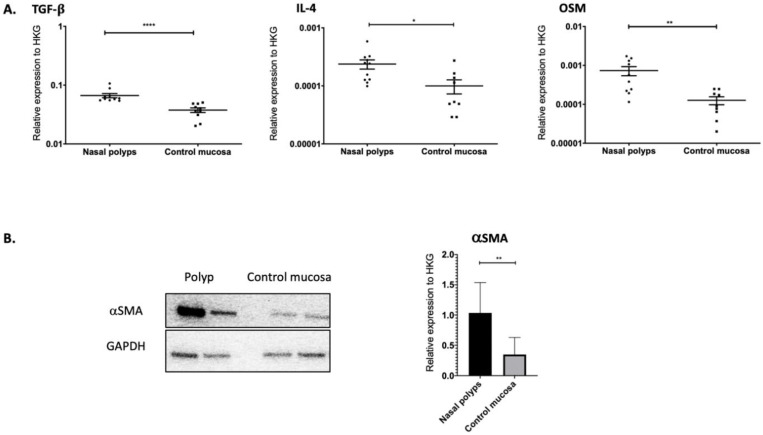
TGF-β1, IL-4 and OSM were overexpressed in nasal polyps (*n* = 13) when compared to noninflammatory nasal mucosa (*n* = 13). TGF-β1, IL-4 and OSM were analyzed by RT-qPCR using GAPDH as housekeeping gene to normalize gene expression (median) (**A**). αSMA was overexpressed in nasal polyps (*n* = 6) when compared to noninflammatory nasal mucosa (*n* = 6). αSMA was analyzed by Western blot using GAPDH as housekeeping gene to normalize gene expression. The image is representative of two experiments. The graph shows the relative expression of each gene compared to the expression of the housekeeping gene (HKG) (*n* = 6) (**B**). **** *p* < 0.0001; ** *p* < 0.005; * *p* < 0.05.

Epithelial cells, fibroblasts and immune cells are able to produce cytokines critical to the genesis of nasal polyps. In nasal polyps and in noninflammatory nasal mucosa, we quantified by RT-qPCR the expression of TGF-β1, OSM and IL-4, which are known to be implicated in fibrotic processes. As shown in Figure 1, TGF-β1 (*p* < 0.0001), OSM (*p* = 0.003) and IL-4 (*p* = 0.013) were overexpressed in nasal polyps compared to noninflammatory mucosa. By a Western blot analysis, we reported the overexpression of αSMA at the protein level in nasal polyps in comparison to noninflammatory mucosa (*p* = 0.004) (Figure 1).

### 2.2. Regulation of Nasal-Polyp-Derived Fibroblasts ECM Components and αSMA by TGF-β1, IL-4 and OSM

**Figure 2:**
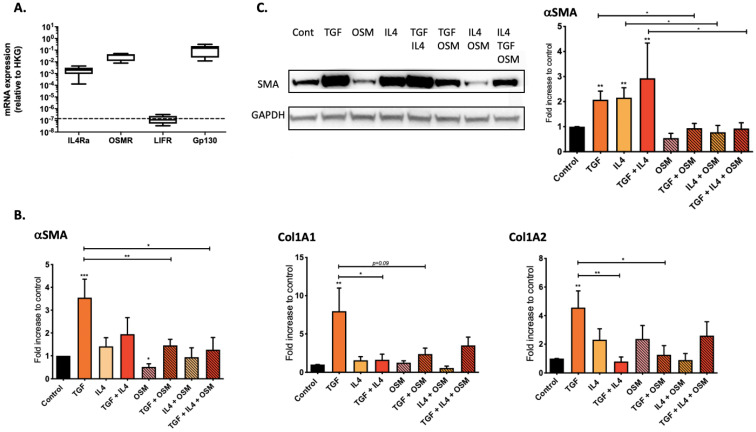
IL-4Rα1, OSMR and gp130, but not LIFR, were expressed in nasal-polyp-derived fibroblasts (*n* = 5). Receptors were analyzed by RT-qPCR using GAPDH as housekeeping gene to normalize gene expression (**A**). Extracellular matrix components and αSMA, mRNA and protein relative expression in nasal fibroblasts cultured with TGF-β1 and/or IL-4 and/or OSM (*n* = 7) were analyzed by RT-qPCR (**B**) and Western blot (**C**) using GAPDH as housekeeping gene to normalize gene and protein expression. The y-axis represents the relative expression normalized to housekeeping gene (HKG). The experiment was repeated four times and images are representative of one experiment. Comparisons are made with the control group (single stars) and between groups (bars) * *p* < 0.05; ** *p* < 0.01; *** *p* < 0.001.

To evaluate the role of the proinflammatory cytokines IL-4 and OSM in the fibrotic process, we evaluated in vitro the expression level of genes coding for ECM components (collagen 1A1 and 1A2) and αSMA (indicator of myofibroblast transition) both at the transcriptional and protein levels in cytokine-stimulated nasal-polyp-derived fibroblasts. We first confirmed the mRNA expression of IL-4Rα1 and type II OSM receptor (composed of OSMR and gp130 chains) but not type I OSM receptor (composed of LIFR and gp130 chains) in nasal-polyp-derived fibroblasts (Figure 2A). Then, we stimulated nasal-polyp-derived fibroblasts with TGF-β1 in the presence or absence of IL-4 and/or OSM. In TGF-β1-stimulated fibroblasts, collagen 1A1 (*p* = 0.004), collagen 1A2 (*p* = 0.004) and αSMA (*p* = 0.0006) mRNA were overexpressed (Figure 2B), as well as at the protein level for αSMA (*p* = 0.002) (Figure 2C). Whereas IL-4 alone had no detectable effect on αSMA, collagen 1A1 and collagen 1A2 expressions at the transcriptional level when compared to unstimulated fibroblasts, αSMA protein expression was enhanced (*p* = 0.002). In contrast, IL-4 counteracted the enhancing effect of TGF-β1 on fibroblasts for collagen 1A1 (*p* = 0.015) and collagen 1A2 (*p* = 0.004) mRNA expression, but not αSMA, both at the transcriptional and protein levels (Figure 2B,C). On the other hand, OSM alone or in combination with IL-4 had no effect when compared to control. However, OSM, in combination with TGF-β1, counteracted the profibrotic effect of TGF-β1, decreasing the expression of collagen 1A1 (*p* = 0.093, not significant), collagen 1A2 (*p* = 0.041) mRNA and αSMA both at the transcriptional (*p* = 0.015) and protein levels (*p* = 0.0260) (Figure 2B,C).

### 2.3. OSM Counteracted TGF-β1 Effects via the Inhibition of Smad3 Nuclear Translocation

**Figure 3:**
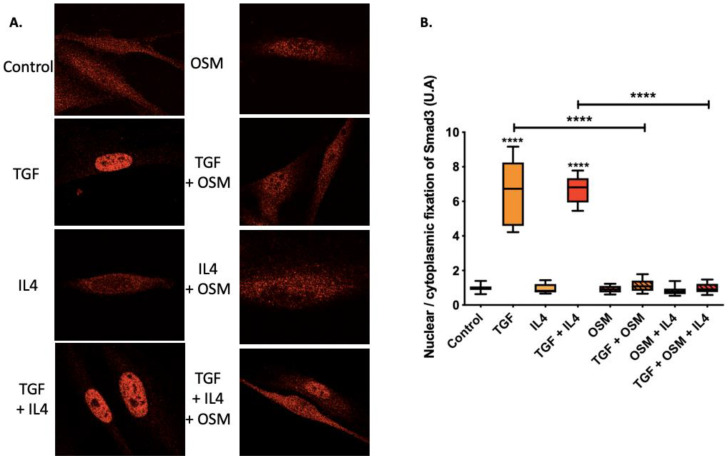
Nuclear/cytoplasmic localization of Smad3 in nasal-polyp-derived fibroblasts cultured with TGF-β1 and/or IL-4 and/or OSM was analyzed under confocal microscopy (**A**,**B**). Pictures are representative of three independent experiments. **** *p* < 0.0001.

To further analyze the effects of OSM and IL-4 on nasal-polyp-derived fibroblasts at the cell signaling level, we studied their potential impact on Smad3 nuclear translocation induced by TGF-β1. Nasal fibroblasts were stimulated with TGF-β1, IL-4 or OSM, alone or in combination. Nuclear or cytoplasmic localization of Smad3 was determined by immunofluorescence and quantified. In TGF-β1-stimulated fibroblasts, the nuclear/cytoplasmic ratio of Smad3 was greater than in unstimulated fibroblasts (*p* < 0.0001) and further addition of IL-4 had no effect. In contrast, OSM reversed the nuclear translocation induced by TGF-β1 (*p* < 0.0001), and the addition of IL-4 to these conditions had no effect (Figure 3).

## 3. Discussion

OSM is a proinflammatory cytokine produced by numerous cells of the immune system such as Th2 lymphocytes, dendritic cells, neutrophils and macrophages. In addition to the proinflammatory effects reported on tissues such as lung [13], joints or liver [14], OSM has the capacity to target skin cells more insensitively than IL-6, as previously described in vitro on primary culture of keratinocytes and in reconstructed human epidermis models [15]. OSM, in combination with other proinflammatory cytokines, plays a major synergistic role in the development of skin inflammation [16,17,18]. In inflammatory diseases, OSM is strongly overexpressed in lesional skin from patients with psoriasis, atopic dermatitis and necrotic angiodermatitis [15,19]. The major role of OSM in the development of chronic inflammatory bowel diseases has also been reported [20]. Our data confirmed that OSM, TGF-β1 and IL-4 were overexpressed in nasal polyps when compared to noninflammatory nasal mucosa. In CRSwNP, OSM has been reported to play a role in the alteration of the nasal epithelium [8]. 

In several diseases associated with fibrosis, OSM plays a regulatory role on fibrosis, associated with pro or antifibrotic roles, depending on the model. In systemic sclerosis, rheumatoid arthritis or interstitial pulmonary fibrosis and other lung diseases associated with fibrosis, OSM has a profibrotic effect [9,10,11]. In a model of pulmonary fibrosis, OSM activates the production of ECM by fibroblasts and inhibits their apoptosis [21]. By contrast, in skin or kidney fibroblasts, OSM reverses the profibrosing effects of TGF-β1. We recently showed that TGF-β1 induces the differentiation into myofibroblasts of dermal fibroblasts from normal skin and hypertrophic scars associated with the exacerbated production of the proteins of the ECM, and OSM, overexpressed in hypertrophic scars, counteracts this effect [12]. Similar observations were made on kidney fibroblasts treated with OSM and TGF-β1 [22].

The role of OSM on fibrosis associated with CRSwNP therefore remained to be studied. In agreement with the central role of TGF-β1 in CRSwNP-associated fibrosis [4], we reported activation and differentiation of nasal fibroblasts into myofibroblasts, as well as the production and deposition of ECM. TGF-β1 signaling includes phosphorylation of Smad2 and Smad3 transcription factors, leading to their nuclear translocation at the origin of the fibrotic cascade [23]. In agreement with Coste et al. and Serpero et al., we confirmed an increase in TGF-β1-induced ECM components and αSMA [24,25]. The IL-4 profibrotic activity described in CRSwNP [6] has been confirmed at the protein level, but not at the mRNA level. It could be due to the kinetics of expression, transient for mRNA, or to post-transcriptional regulatory phenomena. In nasal-polyp-derived fibroblasts, OSM had no effect alone, but possessed an antifibrotic role by counteracting the profibrotic effects of TGF-β1 and IL-4, alone or in combination, on myofibroblast differentiation and ECM component accumulation both at the mRNA and protein levels. The concentration of OSM used (10 ng/mL) was motivated by studies showing that a concentration of 10 ng/mL of OSM allows the optimal effects on fibroblasts and particularly on respiratory-tract-derived fibroblasts [9,21].

**Figure 4:**
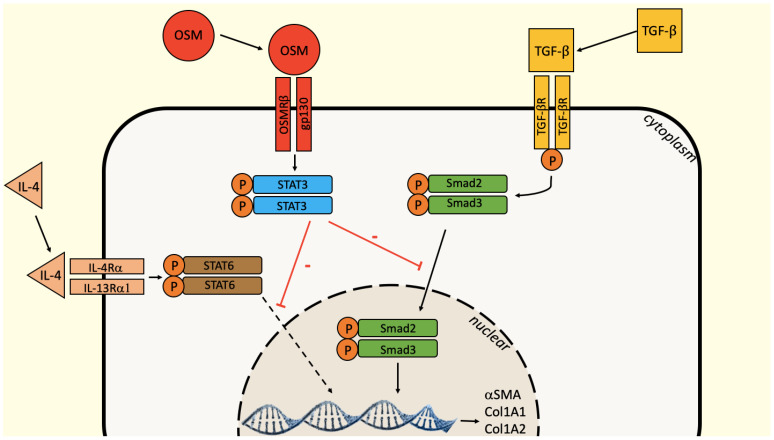
Summary of TGF-β1, IL-4 and OSM integrated effects on fibrosis and differentiation in CRSwNP.

We hypothesize that this inhibition could result from a regulation of the TGF-β1–Smad signaling. The Smad and STAT signaling pathways are involved in a dialogue that modulates fibrotic processes, depending on the model. In hepatic stellate cells, vascular smooth muscle cells or lung-derived fibroblasts, OSM acts synergistically with TGF-β1 and STAT3 cooperates with Smad3 to induce the expression of connective tissue growth factor (CTGF) and stimulate fibrosis [26,27]. TGF-β1 can either directly activate STAT3 via Janus kinase 1 (JAK1), constitutively associated with TGF-βR1, or indirectly via the synthesis of an unidentified protein mediator [28,29]. When OSM antagonizes TGF-β1 activities, such as in kidney fibroblasts, OSM stimulates the STAT3 pathway by phosphorylating STAT3, activating Smad7 at the origin of the inhibition of the nuclear translocation of Smad3 [28]. When fibroblasts from nasal polyps were treated with TGF-β1, the nuclear translocation of Smad3 was increased compared to the control. When OSM was added to TGF-β1, the nuclear Smad3 fixation induced by TGF-β1 was abolished, suggesting an inhibition of the nuclear translocation of Smad3 by OSM, as in the expression of fibrosis factors and αSMA reported above. The profibrotic action of IL-4 is also counteracted by OSM. The IL-4 signaling involves the STAT-6 pathway. To our knowledge, no crosstalk is described between STAT-6 and STAT-3 that could explain this inhibition. This requires further experiments to identify the mechanism of regulation of IL-4 pathway by OSM (Figure 4).

## 4. Materials and Methods

### 4.1. Prospective Clinical Study

This study including 20 patients with CRSwNP was approved by the Institutional Ethics Committee on Human Experimentation of the University Hospital of Poitiers and received the agreement of the French “Ministère de l’Enseignement supérieur et de la Recherche” (DC-2019-3746, December 2019). This study was conducted according to the Declaration of Helsinki principles, and written consent was collected for each patient. Polyps were removed during surgical procedures for CRSwNP. All the patients abstained from oral or topical corticosteroids during one month before the surgery. Control tissues were obtained in 13 patients operated for noninflammatory and nontumoral diseases of the paranasal sinuses. Tissues were immediately placed in culture medium and transported to the laboratory for fibroblast isolation and culture, or frozen in liquid nitrogen before RNA extraction for transcriptional analysis.

### 4.2. Cell Extraction and Culture

Nasal polyp fibroblasts were obtained from surgical samples in patients operated for CRSwNP. Polyps were cut into 2 mm^3^ pieces, placed in a trypsin solution for 30 min (Tryspin-EDTA 0.05% phenol red, Thermo Fisher Scientific, Waltham, MA, USA), passed through a 100 μM corning cell strainer and resuspended in culture medium (Dulbecco’s-modified Eagle’s medium (DMEM) supplemented with 2 mM L-glutamine, 10% fetal calf serum (FCS), 10 μg/mL gentamycine, 10 µg/mL ceftazidime and 2.5 µg/mL amphotericin B (all from Thermo Fisher Scientific, Waltham, MA, USA)). For the quantitative real-time polymerase chain reaction (RT-qPCR) and Western blot analysis, cells were used until the 3rd passage. For immunofluorescence studies, fibroblasts were cultured on 10 mm diameter glass coverslips. For the RT-qPCR, Western blot analysis and immunofluorescence, cells were starved for 24 h in DMEM supplemented with 0.5% FCS before being treated with or without 10 ng/mL recombinant human TGF-β1, IL-4 (PeproTech, Rocky Hill, NJ, USA) or OSM (R&D Systems Europe, Lille, France), alone or in combination for 6 h for immunofluorescence, 24 h for mRNA quantification or 48 h for Western blot analysis.

### 4.3. Quantitative RT-PCR Analysis

Total RNA from nasal fibroblasts was isolated using a NucleoSpin^®^ RNA II kit (Macherey-Nagel, Hoerdt, France) and reverse-transcribed with SuperScript^®^ II reverse transcriptase (Invitrogen, Life Technologies, Carlsbad, CA, USA) according to the manufacturer’s instructions. RT-qPCR was conducted using a Light Cycler-FastStart DNA MasterPlus SYBR^®^ Green I kit and a LightCycler 480 system (Roche Diagnostics, Meylan, France). The reaction components consisted of 1× DNA Master Mix and 0.5 μM sense and anti-sense oligonucleotides purchased from Eurogentec (Eurogentec France, Angers, France) and designed using Primer3 software. Relative RNA expression was determined according to the ΔCT method (relative expression = 2 exp(ΔCT) = 2 exp(CT target − CT glyceraldehyde-3-phosphate dehydrogenase)). 

### 4.4. Western Blotting

After 48 h of stimulation, fibroblasts were lysed with 150 μL of radioimmunoprecipitation assay buffer (RIPA) and protein concentrations were determined by BCP protein assay (Pierce, Thermo Fisher Scientific, Waltham, MA, USA). After separation on a 4–20% SDS-PAGE gel (NuSep, Germantown, Germany), proteins were transferred to nitrocellulose membranes (GE Healthcare, Chicago, IL, USA) by electroblotting. Immunodetection of αSMA and GAPDH was performed by coincubation with rabbit anti-αSMA antibody (Ab) (Novus Biologicals, Centennial, CO, USA) and mouse anti-GAPDH mAb (Novus, clone 2D4A7), followed by coincubation with antirabbit and antimouse IgG peroxidase-conjugated polyclonal Abs (Sigma-Aldrich, St. Louis, MO, USA). Peroxidase activity was detected by chemiluminescence (Luminata HRP substrate from Merck Millipore Burlington, MA, USA) and analyzed using an LAS-3000 imaging system. Signal intensity was measured using ImageJ software. The ratios of αSMA/GAPDH were calculated and are shown in the corresponding figures. 

### 4.5. Immunofluorescence

After 6 h of stimulation, cells were fixed with 4% paraformaldehyde in PBS for 15 min, permeabilized for 10 min in 0.3% Triton X-100 in PBS and stained with 1 µg/mL Hoechst 33342 (Sigma). Cells were incubated for 30 min in PBS containing 1% bovine serum albumin, 1 h with Smad3 mAb (Thermo Fisher Scientific, Waltham, MA, USA), and 1 h with antirabbit RRX conjugated antibody (Jackson ImmunoResearch Laboratories, West Grove, PA, USA) according to the manufacturer’s instructions. Images were generated using an Olympus FV1000 confocal microscope (ImageUP facilities, http://sfa.univ-poitiers.fr/imageup (accessed on 1 January 2015). Nuclear and cytoplasmic localization of Smad3 was quantified using Fiji software. Twenty cells were analyzed for each patient (*n* = 3) and for each condition and the mean value was reported.

### 4.6. Statistical Analysis

Statistical analysis was performed using the Mann–Whitney’s U test. *p*-values less than 0.05 were considered statistically significant. The data are presented as the mean and standard error of the mean.

## 5. Conclusions

We confirmed that OSM, as TGF-β1 and IL-4 are overexpressed in nasal polyps by comparison to noninflammatory nasal mucosa. OSM has the capacity to counteract the profibrotic effect of IL-4 and TGF-β1 by decreasing the inductive accumulation of ECM components and myofibroblast differentiation. In TGF-β1-stimulated fibroblasts, this antagonist activity might be explained by the inhibition of the nuclear translocation of Smad3. 

While hindsight on biotherapies in other autoimmune diseases shows that nonresponders or resistances to treatments are observed, OSM may offer promising strategies for the development of new therapeutic treatments for CRSwNP. However, we should keep in mind that OSM facilitates a delicate tune-up in CRSwNP by inducing inflammation and regulating the associated fibrosis. Is OSM a friend or foe? If low levels could be protective against fibrosis, overproduction could be deleterious by inducing massive inflammation. With a controlled use to avoid its proinflammatory effects, we suggest that OSM could be an efficient tool to protect against fibrosis.

## Data Availability

Data supporting reported results can be found in the Laboratoire Inflammation Tissus Epithéliaux et Cytokines (LITEC), UR15560, Université de Poitiers, 86000 Poitiers.
